# Micro-Organisms and Dental Caries

**Published:** 1884-08

**Authors:** W. D. Miller

**Affiliations:** Berlin.


ARTICLE II.

### MICRO-ORGANISMS AND DENTAL CARIES.

BY DR. W. D. MILLER, BERLIN.

In an address delivered before the Odontological Society of Great Britain, on the 5th of April, 1884*, Messrs. Underwood and Milles presented some views relating to the "Influence of Micro-Organisms in the Production of Caries," which, if established, would tend to show some inaccuracies in the published results of my experiments.

In the first place Messrs. Underwood & Milles failed to produce artificial caries, and in their opinion, as well as in that of others then present, the production of artificial caries is an impossibility.

Inasmuch, however, as I have on various occasions demonstrated preparations of artificial caries here at the Physiological Institute, and to various well-known microscopists in private, the possibility of the production of artificial caries is a fact which does not admit of discussion.

Not alone the softening of the dentine, but the invasion of the micro-organisms, extension of the tubules, breaking down of the basis-substance, melting together of adjacent tubules, the production of round and oval spaces or caverns in the dentine, and lastly, the total disappearance of the

*See *Dental Record* page 225.

dentine—all these may be exactly reproduced by artificial decay.

I shall demonstrate a large number of these preparations at Vevey on the 26th of August next, including the tubules which are shown in the *Independent Practitioner* for May, I can only say to all who doubt the possibility of artificial caries—Come and see. Messrs. Underwood & Milles failed to produce caries for very evident reasons, and, as it happens, those reasons I have given in a paper written three months ago, and published in the May number of the *Independent Practitioner* and in the *Dental Record*, p. 158, etc. In the description of this attempt to repeat my experiments, Messrs. Underwood & Milles say: "The bottle not containing carbolic speedily became the scene of an active growth of micro-organisms, for the greater part micrococci; *a thick felt of this growth formed at the surface of the fluid*.

I have up to the present time isolated five different species of fungi from carious dentine, one of which, from the constancy with which it occurs, may I think, be regarded as a chief factor in caries. Neither this nor any other micro-organism thus far isolated ever forms, in pure cultures, a film upon the surface of the liquid. I am familiar with a felt such as described by Messrs Underwood & Milles; it is not a schizomycete but a blastomycete; it has nothing to do with caries, and, as I stated in the *Independent Practitioner*, when it appears in the culture, the latter may as well be thrown away at once. *I have never succeeded in producing caries where this thick felt was present;* nor could I expect to under such abnormal conditions.

Messrs. Underwood & Milles do not state definitely what the felt consisted of in their case. I should, however, be very much surprised if it were not identical with mine; it was, at any rate, something, which does not and could not well occur in the mouth, and did not occur in the experiments in which I succeeded in producing caries. This experiment, therefore, cannot be accepted as a repetition of mine. Messrs. Underwood & Milles state on page 239* that "the presence of a number of extraneous organisms does in some cases hinder the active growth of others." Perhaps it was this very felt present in their experiments, but not in mine, nor in the mouth, which accounts for their failure. Another great source of error in these experiments lies in the fact that the fungi themselves are rendered inactive by the acid which they have produced; we obtain, consequently, softening of the dentine, but no invasion of fungi. For a full description of this case and the methods employed to avoid, I must refer to the columns of the *Independent Practitioner.*† Within a tooth the enormous extent of surface exposed by the lime salts to the acid prevents its ever becoming so strong as in our cultures.

Again, in the experiments described on pages 235-6 they failed to produce caries, "the putridity of the baths being so offensive" that they abandoned this experiment with relief.

I must confess to not a little surprise that the gentleman should have kept up the experiment at all, for they say themselves (page 251), and I heartily endorse the statement, "we all agree that without some weakening of the enamel by acid nothing in the shape of caries could go on at all," and yet they wasted six months of valuable time in the vain attempt to produce caries in putrid baths which had an alkaline reaction. I have time and again called attention to an experiment which I began may 19th, 1882, over two years ago, in which pieces of dentine have been kept constantly in contact with putrefying substances richly infected from the mouth. Up to the present not a trace of caries or even softening of the sharp points has occurred, although microscopic sections show that here and there a coccus has worked its way into a tubule, which, of course, it might do if the walls of the tubule were of glass—that is no indication of caries.

---

*Transactions of Odontological Society.

†See also *Dental Record* for March, April and May.

It is a fact which I have had abundant opportunity to test, and which every dentist can test for himself, that if we take a freshly extracted *decaying* tooth with pulp intact, thoroughly clean the root from pus, or whatever might produce an odor of putrefaction, remove the *debris* from the cavity, as well as the outer layers of most decomposed dentine and what remains, the truly decaying dentine will have a pure sour smell, never a putrid one. Furthermore, I have during the last six months had constantly in my laboratory from 10-50 cultures of different caries fungi in malt, beef extract, sterilized saliva, calves' broth, blood serum, etc., and have in no case, in pure cultures, observed anything offensive or putrid; especially where sugar is present we always have a thoroughly pure sour, not at all disagreeable smell. It is not strange, then, that no caries occurred in those offensive putrid baths.

Messrs. Underwood and Milles' experiment confirms what I have written in the *Independent Practitioner* for 1883, that the fungi cannot attack sound teeth till they have been acted upon by acids. That this acid is produced largely by the organisms themselves will, I think, be found clearly established in the same journal for February, 1884.

It does not matter how "dirty-mouthed" a man may be, or how much putrefaction may be going on in the oral cavity, that won't produce caries—in fact, the alkaline products of the putrefaction may arrest caries by neutralizing such acid agents as would give rise to it. It is only when the condition is such that the action of the micro-organisms is accompanied by the production of acid and a pure sour smell that caries can be produced.

Mr. Milles (page 241) describes a third attempt to produce caries, but here so important a factor is left out, and the solution used so abnormal, that we would be obliged to predict a failure in the very beginning.

I described at Ostende (1882) a series of experiments showing that fungi taken from carious dentine could not produce softening or decalcification of sound dentine, even when presented to them in the form of powder. In those experiments, however, I made the same mistake that Messrs. Milles & Underwood made in their later attempts to produce caries, viz., I put them under such abnormal conditions that they could not produce the acid necessary to decay.

In the human mouth we have sugar almost always, if not always, present, either taken into the mouth as such, or produced there by the action of the ptyaline of the saliva upon starch.

The fungus, which I consider to be the chief parasitic factor in caries, since I have never failed to find it in carious dentine as well as in saliva, while it has but a slight diastatic action, if any at all, has the power of splitting fermentable sugars, *i. e.*, of converting them into fermentaable varieties. These take place of the simple formulæ:—

$$\underset{\text{Cane Sugar}}{C_{12}H_{22}O_{11}} + H_2O = \underset{\text{Dextrose}}{C_6H_{12}O_6} + \underset{\text{Levulose}}{C_6H_{12}O_6}$$

$$\underset{\text{Fermentable Sugar}}{C_6H_{12}O_6} = \underset{\text{Lactic Acid}}{2\,C_3H_6O_3}$$

We see, consequently, the vast roll played in the mouth by starch and sugar, and in our cultures the latter, at least, should never be omitted, since without it we will obtain but a slight production of acid, and, in most cases, none at all, and without acid we labor in vain to produce caries.

If Messrs. Milles & Underwood had added a little sugar to the blood serum the result would had been different; even then, however the whole difficulty is not overcome. The solution soon becomes so acid as to render the organisms completely inactive, and in liquid media they lie as a sediment on the bottom of the vessel.

I have sought to remedy this by adding pulverized dentine, and, frequently renewing the solution, I can produce softening of the dentine and penetration and expansion of the tubules in three weeks. I do not believe that this can be done in a stinking solution in three years—no, nor in 300 years. Mr. Tomes attributes the failure to produce caries to a lack of oxygen. It is probable, however, that in flask experiments the fungi obtain more oxygen than in the deeper parts of carious dentine. I have shown, moreover (in the *Independent Practitioner* for February, 1884) that these fungi may be successfully cultivated under oil or in flasks, where the atmospheric pressure is only 1-100 the normal, or in flasks which have been deprived of oxygen by an alkaline solution of pyrogallic acid.

In discussing the question of the genetic connection of the different micro-organisms of caries I briefly say that I have never stated that all the micro-organisms found in carious teeth are forms of one fungus; far from it; but that there is one fungus found in the human teeth which produces cocci, bacteria, bacilla, and leptothrix, and that all these forms may be found in concoction.

As stated above, I have, in the last three months, by means of the gelatine culture, isolated four other varieties. I did not suppose that this question would receive any attention from the dental profession; it is, however, a subject which has excited a vast amount of discussion in Germany.

When, therefore, I published an account of this fungus in "Kleb's Archive, 1882," I did so with a full understanding of the question at issue. My preparations were twice demonstrated at the Physiological Institute, and to a great many mykologists in private. Dr. Eidam, of Breslau, assistant of Prof. Cohn, visited me in Berlin, and, after examining my preparations, remarked: "Ya es ist gerade wie sie es beschrieben haben" (Yes, it is exactly as you have described it.) I can also show tubules of carious dentine containing micrococci in one end, bacteria in the middle, and bacilli in the other end, not by any means in all preparations, but in some. There is nothing peculiar in this case: many fungi are known which produce forms morphologically different. At the late Congress of the "Verein fuer Innere Medicin," preparations of the micro-organisms of pneumonia were demonstrated in which cocci were seen united to bacilli. I repeat, therefore, here, the statement made in the *Dental Cosmos*, 1883; "We may have before us a case in which one fungus can produce entirely different forms of development." Although I have already so often demonstrated this fungus I will be ready again to demonstrate it at Vevey to any one who should care to see it.

We come now to the zone of softened dentine. A discussion of this question seems almost unnecessary, since on page 231, Messrs. Underwood & Milles grant the only point for which I have contended when they say: "We do not deny that there is a great probability that the advance guard of micro-organisms may be surrounded, and, to a microscopic extent, only preceded by a softening, the result of the action of these acids, which they themselves produce." That is all I ask for. My object has been always to show the absurdity of the statements of that American school of *non*-investigators who maintain that the organic matter is first devoured by the micro-organisms, thus setting free the lime salts which are *subsequently* washed out mechanically.

I have shown by chemical analyses, which have nothing to fear from repetition, that in a large piece of carious dentine 12–13 of the lime salts were gone, and only 2–5 of the organic matter, thus proving that the decalcification was far in advance of the destruction of the organic matter; that is the point that I wished to establish, and I do not believe that Mr. Underwood differs from me there. Nevertheless, the zone of softened noninfected dentine is a thing which admits of easy demonstration in many preparations.

Some fifteen months ago I sent a number of such preparations to America, which passed freely around from city to city, and were carefully examined by a large number of microscopists. Not one of them, either for or against my views, ever denied the existence of the zone of softened dentine. One of those who have been most opposed to my views, writes: (*New England Journal*, p. 350) "There seems in many cases, at least, a territory of dentine between the portion that is absolutely healthy and the territory where the micro-organisms abound, that is *changed* somewhat, but not absolutely infected." He even attempts to account for this zone by the supposition that the micro-organisms absorb the protoplasm from the fibrils in advance of themselves, thus bringing about the change seen in Dr. Miller's specimens. However absurd the explanation may be, it shows that he recognizes the zone.

Dr. Ross, of Chicapee, writes me that he finds it with ease. Dr. Boynton (*New England Journal*, p. 133) also recognizes it. For my own part I have such an abundance of preparations showing it, that it would be difficult for any one to persuade me that it has no existence. It is when the caries is advancing at right angles to the dentinal tubules that it is particularly pronounced. Take a molar tooth with a medium sized aperature on the grinding surface, from which the caries has extended in all directions under the enamel, break away the walls of the enamel and then with a spoon-shaped excavator be sure that you scoop out all the carious dentine in one piece, cut on the freezing microtome, stain in Fuchsine, and in almost every case large tracts of carious dentine will be found on the sides that are free from organisms. Where the decay is advancing parallel with the tubules, the micro-organisms follow hard upon the softening which they themselves produce; in some cases a few stragglers even may get over the border. At the same time we will often find, even here, not a regular even zone, but large tracts of softened dentine free from organisms.

Messrs. Underwood & Milles' method of testing this question (page 249), is so difficult of carrying out as to render it absolutely impossible. Suppose we have a large cavity containing, say a layer of infected dentine 2 mm. thick, succeeded by a layer of softened, non-infected dentine 1-10 to ¼ mm. thick. Now, if I were compelled on penalty of death to remove all the infected dentine, and to obtain from beneath a piece of the softened, non-infected dentine, without impurifying it by a single coccus from the overlying layers, I could not do it, nor do I believe that any mortal man can do it. I shall be prepared at Vevey to demonstrate these zones, or areas, of softened non-infected dentine on over 300 specimens: there existence is an absolute certainty. I shall there be prepared to cut and mount specimens from large pieces of carious dentine in the presence of the members, and demonstrate the areas cf softened dentine on the same.

I believe, however, that here the difference between us is only imaginary. I hold simply that the fungi cannot attack sound teeth, until they have undergone a certain softening by acids; in other words the decalcification precedes the direct action of the fungi themselves; and if Messrs. Underwood & Milles are of the same opinion, as I judge they are, then there is no occasion to lose so much time in the dispute.

I wish to refer still to the remarks made by Mr. Coleman relative to the change produced in the teeth by exanthemata. This change, as I have frequently pointed out in the American journals, I consider to be entirely independent of micro-organisms; on the other hand it must be sought for in the chemical nature and the nutritive function of the tooth itself.

Why is it that a tooth is so much less soluble in acid, than an equal sized lump of tartar? or why if we mix together the separate constituents of the tooth in the same proportion as they exist in the teeth, do we find the resultant mass so much more soluble than a tooth? The answer is that in the teeth the lime salts form with the organic matter a definite chemical combination, which compared with the lime salts themselves is much more insoluble.

Now this combination we cannot bring about by simply mixing the constituents together; it is a result of the vital force in the tooth itself, and this same vital force which produced the combination can, and, as it seems, does under certain conditions of ill-health dissolve it again, thus rendering the tooth more analogous to a simple mixture of lime salts with organic matter, and of course more easily soluble for acids.

This combination may be stable or unstable; in the former case the teeth are hard, shining, transparent; in the latter they are soft, chalky and opaque. It may at one time be stable, and as the result of some change in the vital or nutritive activity of the tooth, become unstable.

The resistance which a tooth offers to decay will therefore depend, to a certain degree, especially in youth, upon the health of the patient. In old age changes take place much less rapidly in the teeth than in youth, and as a consequence old teeth are not so subject to what we may call spontaneous caries as young teeth. Neither should pulpless teeth be exposed to it, and here is a chance to put this theory to a test. I do not offer the above as the result of experiment, but simply as something which appears to me to offer a possible solution of the question stated.

The fact that micro-organisms play an important part in the production of caries will at present be disputed by very few. It is a point insisted on by many earlier investigators on the subject, by Leler and Rottenstein, by Weil, and many others.

Messrs. Underwood & Milles and myself cannot claim to have done anything more than to have confirmed the views of those investigators, and perhaps to have placed the matter upon a more scientific basis.—*London Dental Record.*

---

ARTICLE III.

